# Aldosterone Inhibits the Fetal Program and Increases Hypertrophy in the Heart of Hypertensive Mice

**DOI:** 10.1371/journal.pone.0038197

**Published:** 2012-05-30

**Authors:** Feriel Azibani, Yvan Devaux, Guillaume Coutance, Saskia Schlossarek, Evelyne Polidano, Loubina Fazal, Regine Merval, Lucie Carrier, Alain Cohen Solal, Christos Chatziantoniou, Jean-Marie Launay, Jane-Lise Samuel, Claude Delcayre

**Affiliations:** 1 Unit 942 INSERM and Université Paris-Diderot, Paris, France; 2 Centre de Recherche Public de la Santé, Luxembourg, Luxembourg; 3 Department of Experimental Pharmacology and Toxicology and University Medical Center Hamburg-Eppendorf, Hamburg, Germany; 4 UPMC, INSERM UMR-S974, CNRS UMR7215, Institut de Myologie, Paris, France; 5 Lariboisière Hospital AP-HP, Paris, France; 6 Unit 702 INSERM and Université Pierre et Marie Curie, Paris, France; Vanderbilt University Medical Center, United States of America

## Abstract

**Background:**

Arterial hypertension (AH) induces cardiac hypertrophy and reactivation of “fetal” gene expression. In rodent heart, alpha-Myosin Heavy Chain (MyHC) and its micro-RNA miR-208a regulate the expression of beta-MyHC and of its intronic miR-208b. However, the role of aldosterone in these processes remains unclear.

**Methodology/Principal Findings:**

RT-PCR and western-blot were used to investigate the genes modulated by arterial hypertension and cardiac hyperaldosteronism. We developed a model of double-transgenic mice (AS-Ren) with cardiac hyperaldosteronism (AS mice) and systemic hypertension (Ren). AS-Ren mice had increased (x2) angiotensin II in plasma and increased (x2) aldosterone in heart. Ren and AS-Ren mice had a robust and similar hypertension (+70%) versus their controls. Anatomical data and echocardiography showed a worsening of cardiac hypertrophy (+41%) in AS-Ren mice (P<0.05 vs Ren). The increase of ANP (x 2.5; P<0.01) mRNA observed in Ren mice was blunted in AS-Ren mice. This non-induction of antitrophic natriuretic peptides may be involved in the higher trophic cardiac response in AS-Ren mice, as indicated by the markedly reduced cardiac hypertrophy in ANP-infused AS-Ren mice for one month. Besides, the AH-induced increase of ßMyHC and its intronic miRNA-208b was prevented in AS-Ren. The inhibition of miR 208a (−75%, p<0.001) in AS-Ren mice compared to AS was associated with increased Sox 6 mRNA (x 1.34; p<0.05), an inhibitor of ßMyHC transcription. Eplerenone prevented all aldosterone-dependent effects.

**Conclusions/Significance:**

Our results indicate that increased aldosterone in heart inhibits the induction of atrial natriuretic peptide expression, via the mineralocorticoid receptor. This worsens cardiac hypertrophy without changing blood pressure. Moreover, this work reveals an original aldosterone-dependent inhibition of miR-208a in hypertension, resulting in the inhibition of β-myosin heavy chain expression through the induction of its transcriptional repressor Sox6. Thus, aldosterone inhibits the fetal program and increases cardiac hypertrophy in hypertensive mice.

## Introduction

Cardiac hypertrophy (CH) is a well-known consequence of chronic arterial hypertension. If untreated, hypertension leads to profound changes of the structural and functional properties of the cardiovascular system, ultimately leading to heart failure. CH is mainly due to hypertrophy of cardiomyocytes that is likely induced by both mechanical and hormonal factors, such as angiotensin II (AngII) and aldosterone [1]. Aldosterone controls the renal sodium reabsorption, thereby playing a major role in the control of plasma volume. In addition, it has emerged in the last years that elevation of plasmatic concentration of aldosterone which occurs in patients with heart failure, is a key-factor of cardiac remodeling [2], [3], [4] and worsening of cardiac properties. The inhibitors of the aldosterone receptor (mineralo-corticoid receptor, MR) have successfully been used in patients with heart failure [5] and are now approved in the clinical treatment of heart failure. However, the mechanisms of the deleterious consequences of aldosterone on cardiac functional properties are not totally understood. Increasing evidence suggests that aldosterone may be a trigger for CH independently of its role in regulation of salt-water balance and blood pressure [6], [7], [8].

**Table 1:** Anatomy and cardiac function.

Groups	WT	AS	Ren	AS-Ren
	n = 6	n = 6	n = 7	n = 6
Systolic blood pressure (mmHg)	86±5	80±2	139±4** £	141±9** £
LVPW (mm)	1.1±0.07	1.2±0.1	1.43±0.04** £	1.4±0.06* £
IVSd (mm)	1.1±0.06	1.1±0.02	1.4±0.05* £	1.4±0.06* £
SF (%)	55.2±0.7	58.3±0.8*	58.3±1.6	61.4±1.3*
*Hormones (plasma)*				
Angiotensin II (fM)	57±3	68±4	88±2*** £	112±4*** £ $
Aldosterone (nM)	0.7±0.02	0.74±0.04	0.7±0.02	1.6±0.1*** £ $
*Hormones (heart)*				
Aldosterone (nM)	8.6±0.5	14.9±0.4**	8±0.4	20.4±0.7*** £ $
*mRNAs (heart)*				
ACE (A.U.)	0.64±0.07	0.54±0.1	0.77±0.06	0.92±0.09* £
*Anatomy*				
BW (g)	29±0.8	30±2	30±0.3	31±0.9
HW (mg)	128±7	149±2	155±8	195±1** $
HW/BW (mg/g)	4.3±0.2	4.8±0.4	5.2±0.2*	6.2±0.2** £ $

Abbreviations: LVPW: Left Ventricular Posterior Wall thickness; IVSd: InterVentricular Septum thickness in diastole; SF: Shortening Fraction; BW: Body Weight; HW: Heart Weight.

* p<0.05.

** p<0.01.

*** p<0.001 vs WT.

£ p<0.05 vs AS.

$ p<0.05 vs Ren.

**Figure 1:**
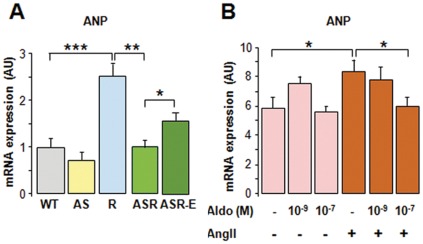
Cardiac hyperaldosteronism inhibits hypertension-induced ANP expression. The expression of ANP was analysed in 9 month-old mice by quantitative RT-PCR analysis: (**A**) in ventricles of control mice (WT, AS), of Ren mice and AS-Ren mice untreated or treated with eplerenone (100 mg/kg/day) for 30 days, (**B**) in NMCM stimulated by 10^−5 ^M AngII in the presence or absence of aldosterone (10^−9^ M or 10^−7^ M) (data are mean of 3 independent experiments). **Abbreviations**: WT: wild-type mice, AS: aldosterone-synthase overexpressing mice, R: renin-overexpressing mice, ASR: AS and R crossed-mice (see details in methods); Aldo: aldosterone; AngII: angiotensin II; Eple: eplerenone treatment; AU: Arbitrary Units; n = 10–12/group. *p<0.05; **p<0.01; ***p<0.001.

To prevent cardiac failure, it is necessary to identify precisely the molecules that regulate the hypertrophic program. In hypertension-induced CH, cardiomyocytes undergo important molecular changes that initially allow the adaptation to increased wall stress. These changes involve the re-expression of a fetal genetic program identified by the induction of natriuretic peptides (NPs), β-myosin heavy chain (β-MyHC), and α- skeletal actin [1]. Cardiac hypertrophy is in part activated by multiple signalling molecules: the calcineurin phosphatase, the serine/threonine protein kinase AKT, the extracellular regulated MAP kinase (ERK), and several transcription factors like NFAT (nuclear factor of activated T-cells), GATA4, MEF2 (myocyte enhancer factor 2), SRF (serum response factor) and p-CREB (phosphorylated cAMPresponse element binding) acting on different target genes [9], [10]. In addition many genes are under fine post-transcriptional control by microRNAs (miRNAs). The *Myh6* gene encodes α-MyHC and the intronic miR-208a. Similarly, the *Myh7* gene encodes β-MyHC and miR-208b. MiR-208a inhibits the Sox6 factor, which inhibits the expression of both β-MyHC and miR-208b [11], [12].

This work aimed to study the role of aldosterone in the mechanisms and signalling pathways regulating CH in transgenic hypertensive mice. Our results indicate that elevated levels of aldosterone in the heart inhibit NPs and β-MyHC expression through original mechanisms.

**Figure 2:**
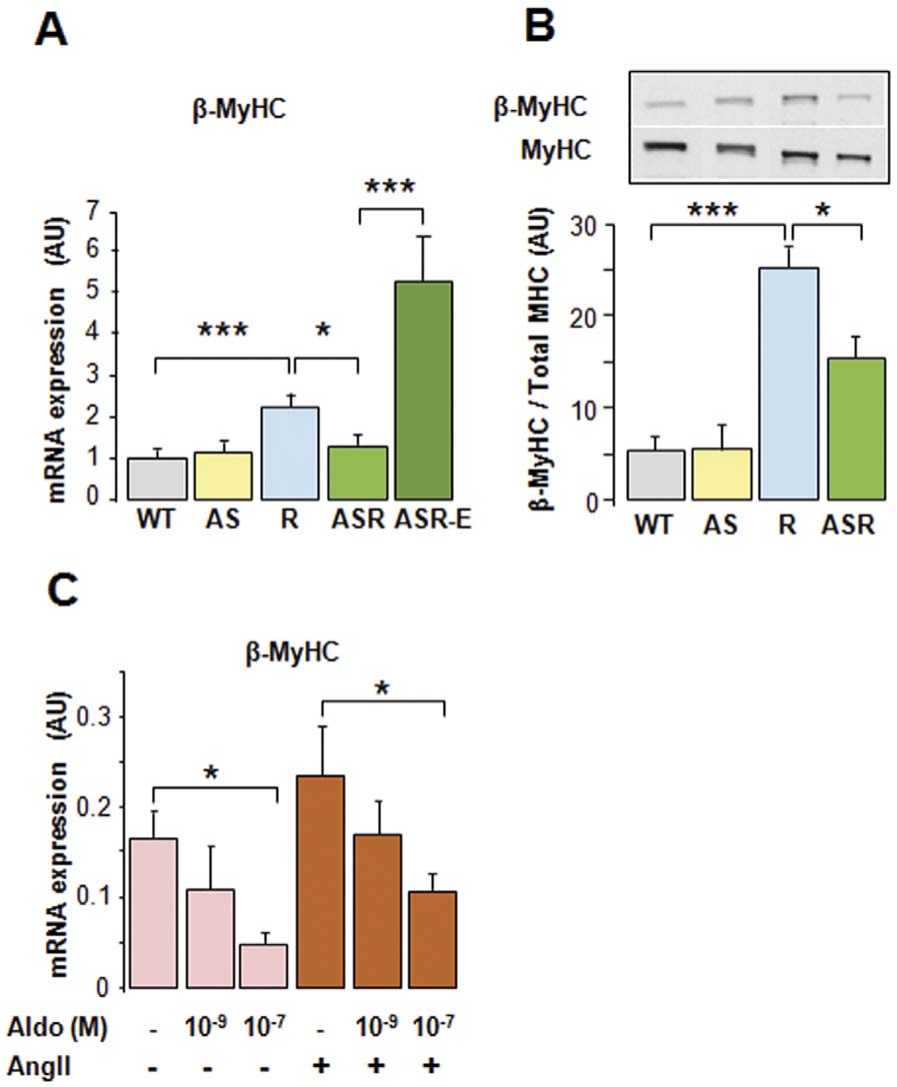
Cardiac hyperaldosteronism inhibits hypertension-induced β-MyHC expression. The expression of β-MyHC was analysed in 9 month-old mice by: (**A**) quantitative RT-PCR in ventricles of control mice (WT, AS), of Ren mice and AS-Ren mice untreated or treated with eplerenone (100 mg/kg/day) for 30 days, (**B**) quantification of β-MyHC and total MyHC protein levels in ventricles after immunoblot (representative signals are shown), (**C**) quantitative RT-PCR in NMCM stimulated by 10^−5 ^M AngII in the absence or presence of aldosterone (data are mean of 3 independent experiments), Abbreviations and symbols as in Figure 1. n = 10–12/group.

## Materials and Methods

### Animals

All experimentations were conducted in agreement with accepted standards of animal care, as outlined in the NIH Guide for the Care and Use of Laboratory Animals and were approved by the local ethics committee (Ethics Committee for Animal Experimentation Lariboisiere-Villemin of the School of Medicine Paris 7, Paris, France). The local ethics committee specifically approved this study (n°2010-12-02). Double transgenic mice (AS-Ren) were created by crossing RenTgKC female with AS male mice. RenTgKC mice backcrossed in the genetic background 129 SV overexpress a synthetic renin cDNA (Ren-2 and Ren-1d genes) in liver and develop early a sustained hypertension [13], [14]. AS male mice were raised by our laboratory in a FVB background to over-express the aldosterone-synthase (AS) gene in cardiomyocytes. To this end, the AS gene expression was under the control of an alpha-myosin heavy chain promoter sequence inserted in the transgene. AS mice have a normal cardiac phenotype despite a 2-fold higher intracardiac aldosterone concentration [15], except a BKCa channel dependent coronary dysfunction [16]. AS-Ren mice of F1 generation were used in all experiments. All mice were viable and only male were used. WT and AS littermates were used as controls. 6 or 9 month-old mice were sacrificed with an overdose of pentobarbital. The hearts were arrested in diastole by an intravenous injection of saturated KCl, they were quickly removed, weighed and cut transversely at the ventricles equator. The upper parts of the heart were mounted, frozen in isopentane precooled with liquid nitrogen, and kept at −70°C until use, as previously described [17].

**Figure 3:**
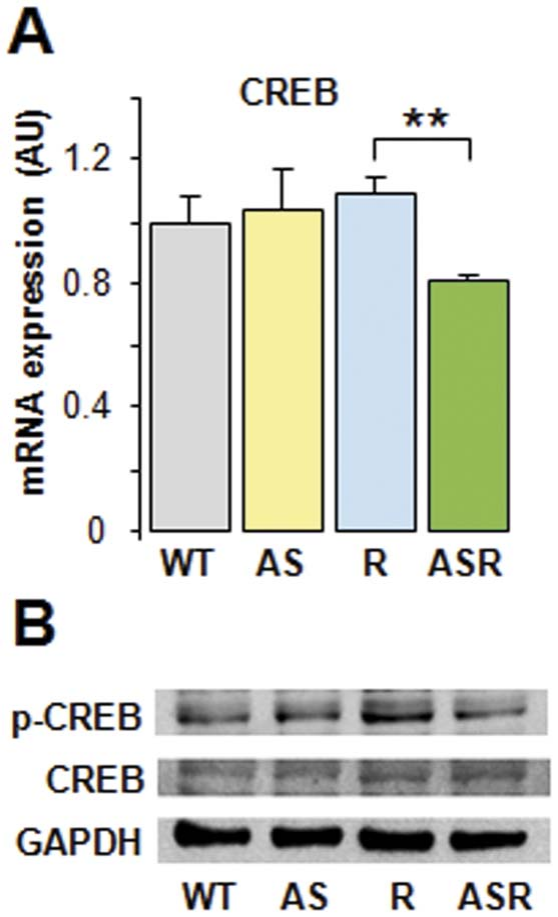
Effect of aldosterone on CREB expression. (**A**): quantitative RT-PCR analysis of CREB in 9 month-old mouse ventricles and (**B**) representative immunoblots of p-CREB, CREB and GAPDH levels in ventricle protein extracts. Abbreviations and symbols as in Figure 1.

### Eplerenone Treatment

The mineralocorticoid receptor (MR) antagonist eplerenone (Tocris, Bristol, UK) was administered in food to mice at 100 mg/kg/day as previously described [18], in 6 and 8 month-old mice for 10 and 30 days before sacrifice, respectively.

**Figure 4:**
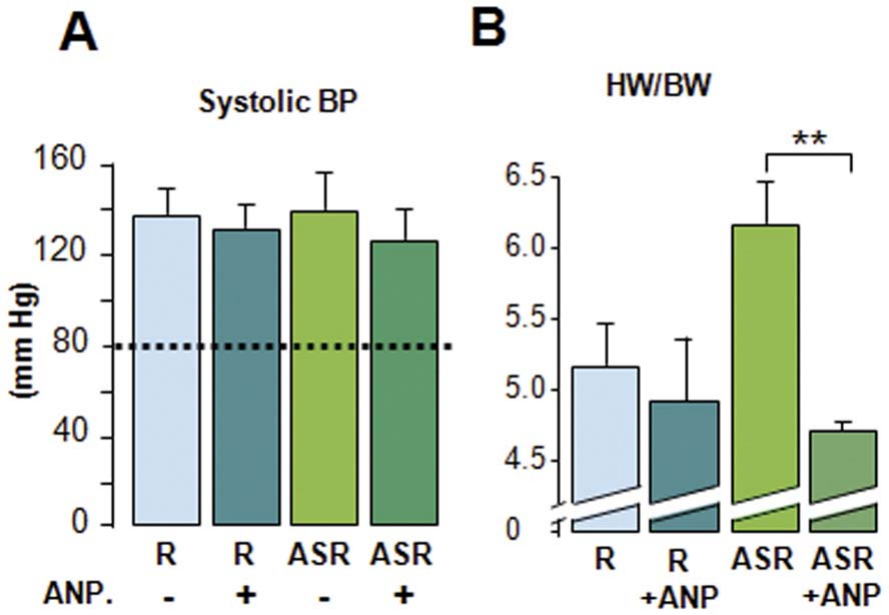
ANP infusion reduces cardiac hypertrophy. (**A**): systolic blood pressure and (**B**) heart weight/body weight ratio (HW/BW) of 9 month-old Ren and AS-Ren mice treated with ANP for 1 month. Abbreviations and symbols as in Figure 1. n = 5–6/groups.

### ANP Infusion

After isoflurane (2%) anaesthesia, 8-month old Ren and AS-Ren mice were implanted subcutaneously with osmotic minipumps (Alzet, 2004) delivering 50 µg/kg/day ANP (Sigma-Aldrich, Saint Louis, MO) for 1 month. These animals were thus named “9-month old mice” in the text. Since sham pump implantation was devoid of any effect on blood pressure and cardiac hypertrophy, non-implanted Ren and AS-Ren mice were used as controls to the ANP-infused mice.

**Figure 5:**
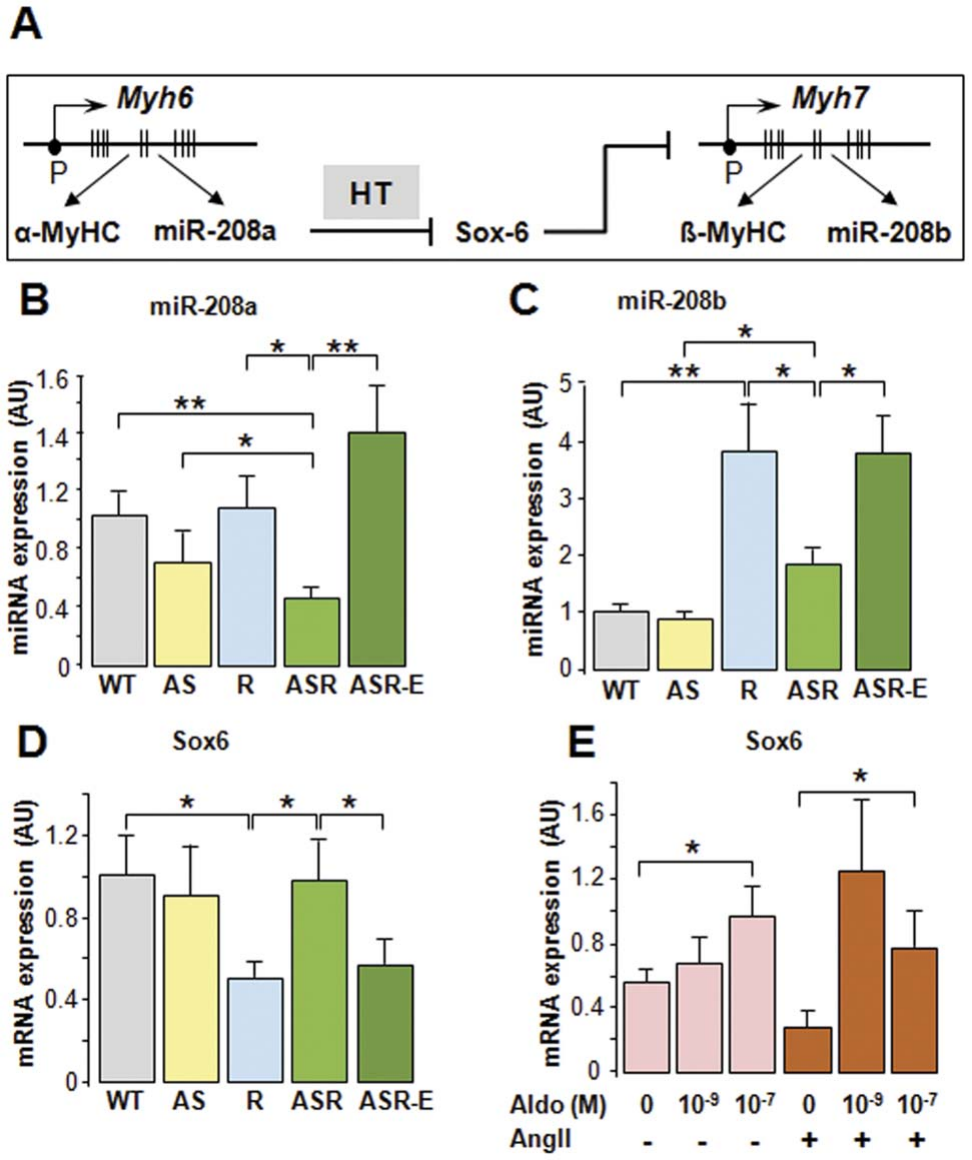
Effect of combined cardiac aldosterone and hypertension on cardiac miR-208 and Sox6 expression. (**A**): scheme of the interplay of miR-208a and -208b on the regulation of *Myh7* expression in hypertensive heart, as drawn from literature data. (**B-D**): quantitative RT-PCR analysis of miR-208a, Sox6 and miR-208b expression in 9 month-old WT, AS, R and AS-R mice and in AS-R eplerenone treated mice (100 mg/Kg/day) for one month. (**E**): quantitative RT-PCR analysis of Sox6 expression in NMCM stimulated by 10^−5 ^M AngII in the presence or absence of aldosterone (10^−9^ M or 10^−7^ M). Abbreviations and symbols as in Figure 1; HT: hypertension; n = 10–14/group.

### Blood Pressure

Systolic blood pressure was measured in unanesthetized mice by the tail-cuff method (Kent Scientific Corporation, Torrington, USA) after daily accustomization during 2weeks before measurements. Only animals that did not display signals of stress and that showed stable and reproducible values of blood pressure for at least three consecutive days were considered for blood pressure measurements. Ten measurements from each mouse were taken at two minutes intervals then a mean value was determined.

### Echocardiography

Echocardiographies were performed on Ketamin (Virbac, 100 mg/Kg) anesthetized animals, in a blinded fashion using a GE Vivid-7 machine (General Electric Medical Systems, Paris, France) equipped with an 8–14 MHz linear transducer. The thickness of the left ventricle LV and fractional shortening were measured as in [19].

### Aldosterone and AngII Measurements

Blood was collected during the sacrifice and plasma was isolated by centrifugation. Aldosterone and AngII plasmatic and cardiac concentrations were measured by radioimmunoassay (RayBiotech company, Cayman chemical company, respectively) according to the manufacturer’s instructions.

### Immunofluorescence Analysis

Immunostaining was performed on cryostat sections fixed with 4% PFA. Identification of cardiomyocytes was performed with mouse vinculin antibody (Sigma Aldrich, Saint Quentin Fallavier, France, 1/250), as previously described [18]. A Leica Camera equipped with a fluorescent Leica DMR (Leica Microsys tems, Rueil Malmaison, France) allowed the fluorescence detection.

### RNA Extraction and PCR

RNA was isolated using the RNeasy Mini Kit (Qiagen, Courtaboeuf, France) according to the manufacturer’s instructions and reverse transcribed using Ready-to-go You-Prime First-Strand Beads kit (GE Healthcare) with 2 µg of RNA and 0.5 µg of oligo dT (Invitrogen, Saint Aubin, France). Finally, real-time amplification was performed with Mastercycler Realplex2 (Eppendorf, Le Pecq, France) using the MESA BLUE qPCR MasterMix Plus for SYBR® Assay No ROX (Eurogentec, Angers, France). mRNAs data were normalized by the abundance of glyceraldehyde-3-phosphate dehydrogenase (GAPDH) mRNA for each sample and expressed as the relative change compared with the control sample. Primers are listed in Supplemental Table S1.

### MicroRNAs Analysis

Total RNA was extracted from frozen tissue using the mirVana kit (Life Technologies, Saint Aubin, France) according to the manufacturer’s instructions without enrichment for small RNAs. Potential genomic DNA contamination was eliminated using DNA-free kit (Qiagen). RNA (150 ng) was used per 20-µL reaction to generate cDNA using the miScript reverse transcription kit (Qiagen). Quantitative RT-PCR was performed using 30 ng cDNA with specific miScript primer sets and miScript SYBR Green PCR kit (Qiagen). Quantitative measurements of miRNA levels were normalized for isolation efficiency using a mix of 3 spiked-in synthetic Caenorhabditis elegans miRNAs (Qiagen), lacking sequence homology to human miRNAs, as described previously [20]. Oligonucleotides were spiked into the samples during RNA isolation after tissue incubation with the provided lysis solution. In addition, results were corrected using the endogenous miR-16.

### Western Blot Analysis

Heart tissue was homogenized in a lysis buffer (50 mM Tris HCl pH 7.4, 5 mM EDTA, 100 mM NaCl, 0.2 mM sodium vanadate, 50 mM sodium fluoride, 40 mM β-glycerophosphatase), with 1% Triton ×100 for myofibrils preparation. Lysates were incubated 1 h on ice before clearing by centrifugation (12000 rpm, 4°C, 20 min). Pellets dissolved in 1% SDS were quantified with Pierce BCA Protein Assay Kit (Pierce Biotechnology, USA). Proteins (0.4 µg) were fractionated on a 4–20% polyacrylamide gel (Invitrogen) and transferred onto nitrocellulose membrane. After blocking, nitrocellulose blots were incubated with mouse anti-β-MyHC (Sigma-Aldrich, Saint Quentin Fallavier, France, 1/2000) or mouse anti-MyHC (Sigma-Aldrich, 1/5000) overnight at 4°C. A secondary antibody coupled to horseradish peroxidase (1/5000, Sigma) was then applied for 2 h at 21°C and detection was performed with enhanced chemiluminescence (ECL Plus, GE Healthcare). Chemiluminescence was measured using a LAS 3000 and MultiGauge V2.02 software (Fuji, Courbevoie, France).

### In vitro Studies

Isolation of neonatal mouse cardiomyocytes (NMCM) was performed as described [21]. After 72 h of culture, plated cardiomyocytes were starved (0.2% Serum) and pre-treated with aldosterone (10^−9^ or 10^−7^ M) overnight (37°C, 5% CO_2_). Then, 10^−5^ M AngII was added and RNA was extracted after 24 h.

### Statistical Analysis

All results are expressed as means ± SEM. Following ANOVA analysis, a Student’s t test with Bonferroni correction was performed except otherwise indicated. p<0.05 was considered as statistically significant. The significance is indicated by a horizontal line between the two groups of interest.

## Results and Discussion

### Effects of Cardiac Hyperaldosteronism and/or Hypertension on Cardiac Hypertrophy

The study was done on 6 and 9 month-old mice in order to study the development of cardiac hypertrophy. Blood pressure was already increased at 6 month of age in Ren and AS-Ren mice, and further increased at 9 months when systolic pressure reached +62% and +76% in Ren and AS-Ren mice when compared to WT and AS mice, respectively (Table 1). Plasmatic AngII concentration was higher in Ren mice and further enhanced in AS-Ren. The cardiac ACE mRNA level and plasma aldosterone concentration were increased in AS-Ren mice only. Cardiac aldosterone concentration was elevated in AS mice as already observed in this strain [15], and its level was enhanced in AS-Ren mice (Table 1). The increase in plasmatic AngII level in AS-Ren relative to Ren might be secondary to the aldosterone-induced ACE upregulation in the heart [22], [23]. These results illustrate the existence of a vicious cycle between peripheral renin-angiotensin system and cardiac aldosterone synthase, which likely results in both an increase of AngII plasma level and a leak of cardiac aldosterone in the blood stream. Both the echocardiographic (Left Ventricular Posterior Wall and diastolic InterVentricular Septum thicknesses) and anatomic (heart weight/body weight ratio) data of hypertensive mice indicated signs of concentric cardiac hypertrophy. However, the cardiac hypertrophy of AS-Ren mice was markedly higher than that of Ren mice (+20% vs. Ren mice; p<0.05) (Table 1, Supplemental Figure S1). This difference of hypertrophic response despite the similar levels of hypertension in Ren and AS-Ren mice indicates that the pro-trophic effect of aldosterone is potentiated by hypertension. This result is in agreement with experimental [24] and clinical [25], [26] studies using MR antagonists. This is also in line with the strong correlation between left ventricle (LV) mass index and serum aldosterone levels described in patients with essential hypertension [27], [28]. The evidence of a trophic effect of aldosterone was strengthened by the results of an eplerenone treatment, which decreased significantly the AS-Ren heart weight/body weight ratio (−10% vs. non treated AS-Ren mice) without any change of blood pressure after 10 days (Supplemental Figure S2). Furthermore, a partial prevention of cardiac hypertrophy by 1month of eplerenone treatment in AS-Ren mice was detected by the echocardiographic (LVPW) and anatomical data (HW/BW) (Supplemental Table S2). This result was in agreement with the very recent data of Fraccarollo et al showing that cardiomyocyte-specific MR ablation prevents the development of cardiac hypertrophy after myocardial infarction [29].

### Hyperaldosteronism Regulates Fetal Genes Reexpression in Heart

As expected in such a hypertension model, the expression of the fetal genes (*Nppa* and *Myh7,* coding for ANP and β-MyHC, respectively) was robustly induced in Ren mice (x2.5, x2.2; p<0.001 respectively; Figures 1 and 2). Interestingly, this induction was markedly inhibited in AS-Ren mice (−60%, −43% vs. Ren; p<0.05). Similarly to its mRNA, the β-MyHC protein level was increased in Ren and AS-Ren hypertensive mice (Figure 2B). However, this increase was partly blunted by cardiac hyperaldosteronism (−40% vs. Ren mice; p<0.05), in line with transcriptional analysis. These results contrast with others suggesting that aldosterone favours the cardiac fetal gene expression, likely through activation of calcineurin pathway [30]. This discrepancy might be due to an increased type I angiotensin receptor activity in vivo [31] or to experimental conditions that would differ for aldosterone concentration or time points. In cultured NMCM, aldosterone alone inhibited the expression of β-MyHC mRNA (Figure 2C). When the cardiomyocytes were stimulated with AngII, aldosterone dose-dependently inhibited the expression of ANP and β-MyHC mRNAs (Figures 1B and 2C). In vivo, the aldosterone effect was reversed in presence of eplerenone (Figures 1A and 2A). Altogether, these data indicate that aldosterone inhibits β-MyHC and NPs expression via the MR signaling pathway.

*Act1* encoding α-skeletal actin is one to the fetal genes up-regulated during an hypertension-induced cardiac hypertrophy [32]. Its expression requires the serum response factor (SRF) associated with GATA4 [33]. Of note, the mRNA expression of *Act1* and SRF increased in a similar manner in both Ren and AS-Ren groups (Supplemental Figure S3A-B), indicating a reactivation of SRF pathway in the hypertensive hearts independently of aldosterone.

### Implication of p-CREB Signaling in Aldosterone Inhibition of ANP Expression

The cAMP-CREB pathway, which is involved in the transcription of *Nppa* [34], is inhibited by aldosterone [35], [36]. We observed that aldosterone inhibited CREB transcription and phosphorylation in AS-Ren hearts (Figure 3), suggesting that aldosterone impedes ANP transcription by inhibiting CREB activation.

Natriuretic peptides (NPs) being known to counteract the development of hypertrophic process [37], the observations that eplerenone treatment upregulated NPs and inhibited the hypertrophic process in AS-Ren prompted us to test if ANP might reverse the development of cardiac hypertrophy in these mice. Thus, ANP minipumps were implanted in 8 month-old AS-Ren mice for one month. This chronic ANP infusion had no effect on blood pressure in any group (Figure 4A), but it decreased the cardiac hypertrophy only in AS-Ren mice whereas it had no action on the heart weight of Ren animals (Figure 4B). Consequently, it emerged that aldosterone worsened the development of cardiac hypertrophy through a MR-dependent inhibition of ANP expression.

### Regulation of miRNAs Expression by Cardiac Hyperaldosteronism Combined with Arterial Hypertension

As outlined in the Introduction section and in Figure 5A, the expression of *Myh7* is controlled by miR-208a and the Sox6 transcription factor [11], [12]. As expected in the experimental groups, the expression of miR-208b (x3.8; p<0.01 vs. control mice; Figure 5D) mirrored that of β-MyHC mRNA (Figure 2A). Indeed, the maintained expression of miR-208a and the strong induction of miR-208b mRNA in Ren mice (Figure 5B and C) was in agreement with the low Sox6 mRNA level (−50%, p0.05 vs. control mice, Figure 5D). Interestingly, in AS-Ren mice the increase of miR-208b transcription that was observed in Ren mice was significantly inhibited (−60% vs. Ren). Thus, the maintained level of Sox6 transcription observed in AS-Ren mice (+50%; p<0.05 vs. Ren) is likely involved in *Myh7* repression. The specific inhibition of miR-208a observed in AS-Ren mice (−58% vs. WT and Ren mice; −35% vs. AS mice) (Figure 5B) is consistent with the Sox6 and α-MyHC mRNA profile. In addition, aldosterone increased Sox6 expression in NMCM with or without AngII (Figure 5E). Hence, the cardiac hyperaldosteronism inhibits miR-208a expression, allowing a sustained Sox6 expression, which in turn inhibits the *Myh7* transcription.

Furthermore, in AS-Ren mice the expression profiles of miR-208a, Sox6, miR-208b (Figure 5 A, C and B) and of β-MyHC (Figure 2D) were reversed by eplerenone (+250%, −50%, +221%, +242%, respectively, vs. untreated AS-Ren) indicating involvement of the MR pathway.

### Conclusions

Using combined hypertension and hyperaldosteronism and both in vivo and in vitro studies, it is demonstrated for the first time that aldosterone, via its mineralocorticoid receptor, represses the expression of ANP and ß-MyHC in a pathological context, thereby amplifying cardiac hypertrophy. Our data provide important information for the understanding of aldosterone-MR implication in the cardiac hypertrophy machinery.

## Supporting Information

Figure S1 **Aldosterone worsens cardiac hypertrophy in hypertension.** Representative cardiomyocyte hypertrophy assessed by vinculin immunolabeling on LV sections of 9 month-old control (WT, AS) and hypertensive (R, AS-R) mice. External membrane of cardiomyocytes is highlighted in red to show the increase of cell size in hypertension. Fluorescent area (*) indicates en face intercalated disc. Bar: 100 µm. Abbreviations: WT: wild-type mice, AS: aldosterone-synthase overexpressing mice, R: renin-overexpressing mice, ASR: AS and R crossed-mice.
(TIF)

Figure S2 **Prevention of aldosterone effects by eplerenone in 6 month-old AS-Ren mice.** (A): systolic blood pressure and (B) Heart weight/body weight ratio (HW/BW) of 6 month-old Ren and AS-Ren mice treated with eplerenone (Eple, 100 mg/Kg/day) for 10 days. In panel A, the horizontal dotted line represents the systolic blood pressure in normotensive mice. (C-E): quantitative RT-PCR analysis of ANP, β-MyHC, miR-208a, Sox6 and miR-208b expression in 6 month-old AS-Ren eplerenone-treated and untreated mice. Abbreviations: HW/BW: heart weight/body weight ratio; R: renin-overexpressing mice, ASR: AS and R crossed-mice; AU: Arbitrary Units. Eple: eplerenone. n = 6–8.
(TIF)

Figure S3 **Effect of AngII and aldosterone on cardiac phenotype.** RT-PCR quantification of α skeletal actin, SRF, α-MyHC, BNP and cardiac α-actin mRNAs level in 9 month-old mice. (6 - 10 in each group). Abbreviations: WT: wild-type mice, AS: aldosterone-synthase overexpressing mice, R: renin-overexpressing mice, ASR: AS and R crossed-mice; AU: Arbitrary Units.
(TIF)

Table S1 **Sequence of primers used for PCR.**
(DOC)

Table S2 **Effect of eplerenone on hemodynamic and anatomical indexes of 9-month old mice.**
(DOC)
