# Supplementary figures and images for: Aldosterone Inhibits the Fetal Program and Increases Hypertrophy in the Heart of Hypertensive Mice
Source: PLoS One. 2012 May 30;7(5):e38197. doi: 10.1371/journal.pone.0038197 (PMC3364229; doi:10.1371/journal.pone.0038197)

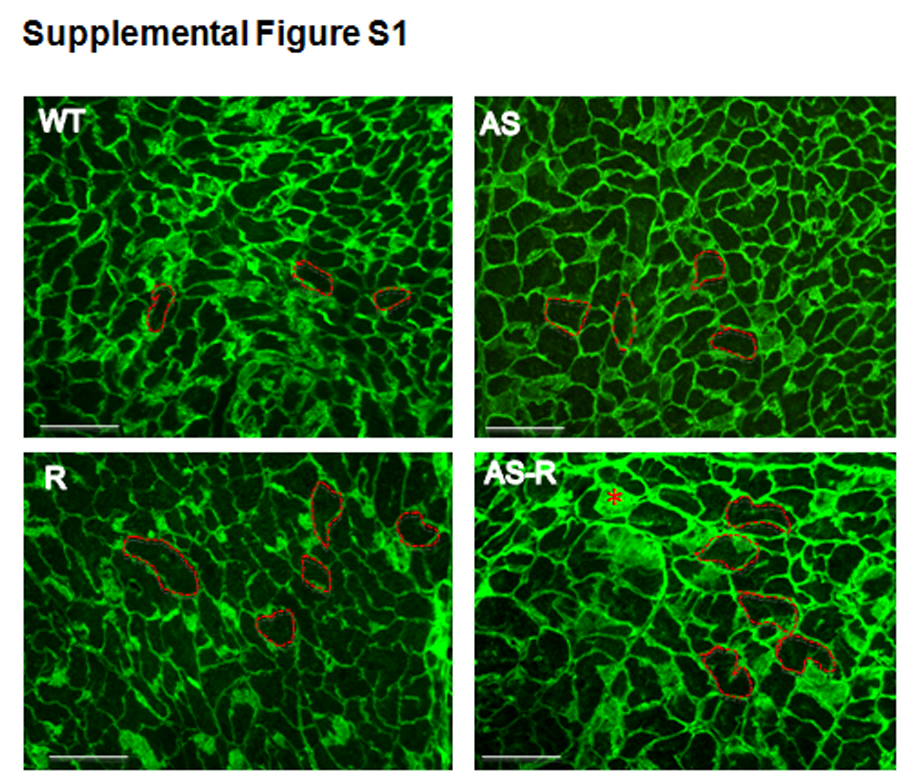

Supplement: Figure S1 — Aldosterone worsens cardiac hypertrophy in hypertension. Representative cardiomyocyte hypertrophy assessed by vinculin immunolabeling on LV sections of 9 month-old control (WT, AS) and hypertensive (R, AS-R) mice. External membrane of cardiomyocytes is highlighted in red to show the increase of cell size in hypertension. Fluorescent area (*) indicates en face intercalated disc. Bar: 100 µm. Abbreviations: WT: wild-type mice, AS: aldosterone-synthase overexpressing mice, R: renin-overexpressing mice, ASR: AS and R crossed-mice. (TIF) [file pone.0038197.s001.tif]

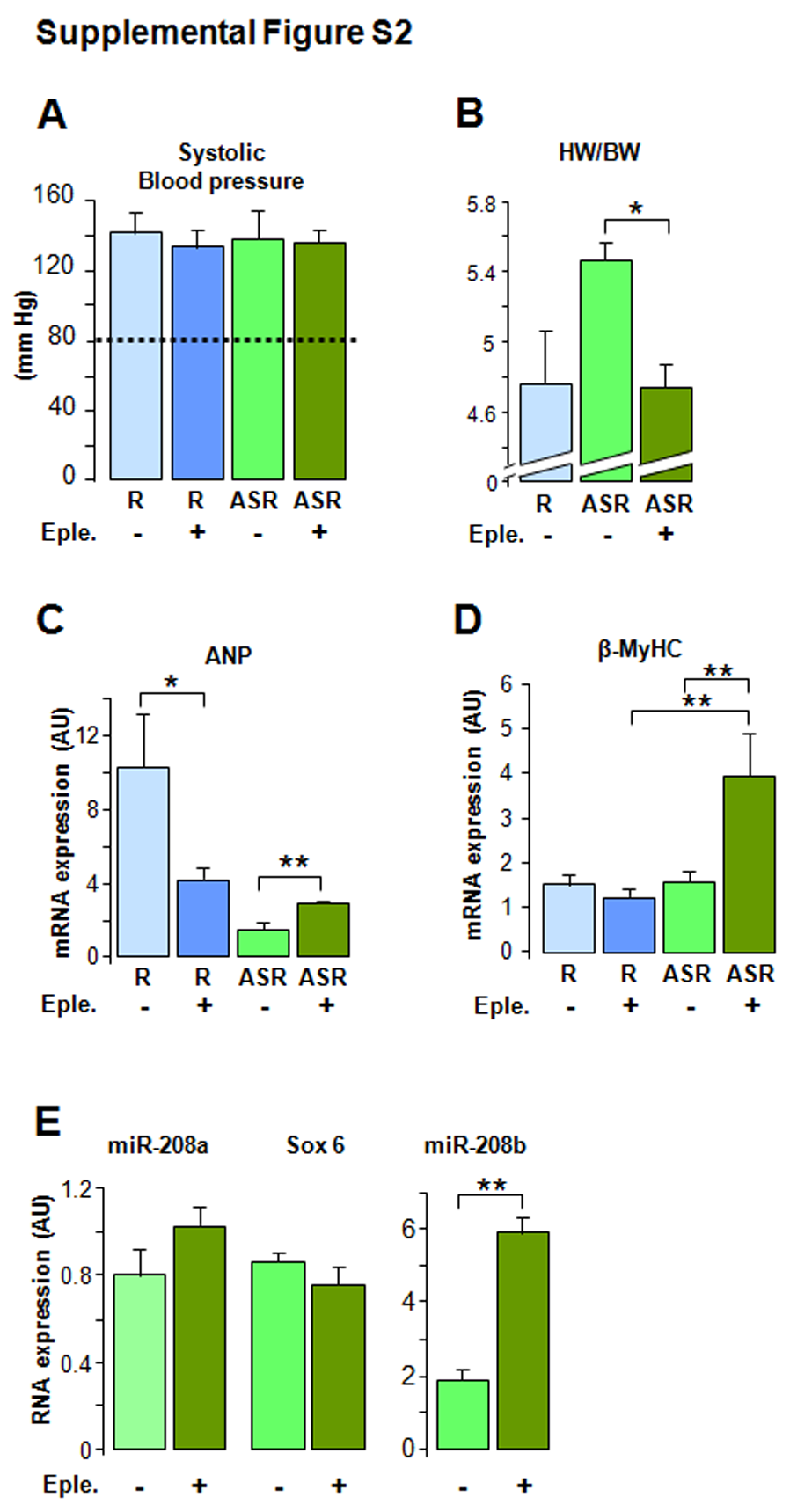

Supplement: Figure S2 — Prevention of aldosterone effects by eplerenone in 6 month-old AS-Ren mice. (A): systolic blood pressure and (B) Heart weight/body weight ratio (HW/BW) of 6 month-old Ren and AS-Ren mice treated with eplerenone (Eple, 100 mg/Kg/day) for 10 days. In panel A, the horizontal dotted line represents the systolic blood pressure in normotensive mice. (C-E): quantitative RT-PCR analysis of ANP, β-MyHC, miR-208a, Sox6 and miR-208b expression in 6 month-old AS-Ren eplerenone-treated and untreated mice. Abbreviations: HW/BW: heart weight/body weight ratio; R: renin-overexpressing mice, ASR: AS and R crossed-mice; AU: Arbitrary Units. Eple: eplerenone. n = 6–8. (TIF) [file pone.0038197.s002.tif]

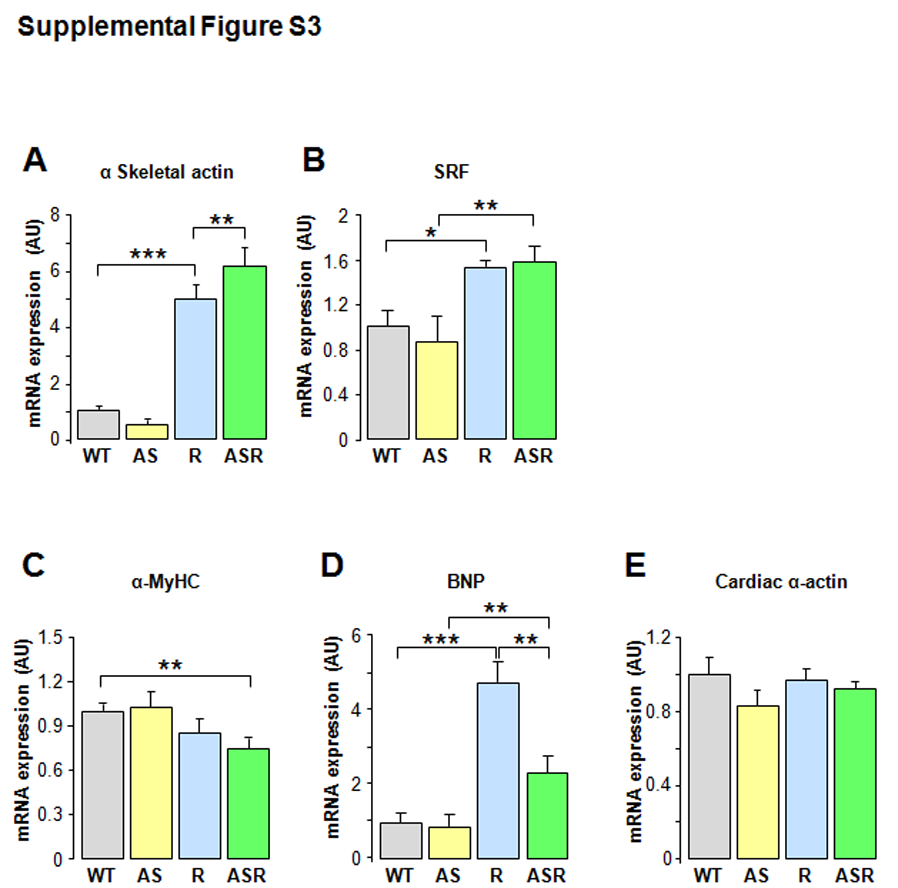

Supplement: Figure S3 — Effect of AngII and aldosterone on cardiac phenotype. RT-PCR quantification of α skeletal actin, SRF, α-MyHC, BNP and cardiac α-actin mRNAs level in 9 month-old mice. (6 - 10 in each group). Abbreviations: WT: wild-type mice, AS: aldosterone-synthase overexpressing mice, R: renin-overexpressing mice, ASR: AS and R crossed-mice; AU: Arbitrary Units. (TIF) [file pone.0038197.s003.tif]
