# Supplementary material for: Aldosterone Inhibits the Fetal Program and Increases Hypertrophy in the Heart of Hypertensive Mice
Source: PLoS One. 2012 May 30;7(5):e38197. doi: 10.1371/journal.pone.0038197 (PMC3364229; doi:10.1371/journal.pone.0038197)
Supplement: Table S1 — Sequence of primers used for PCR. (DOC) [file pone.0038197.s004.doc]

**Supplemental Table S1**

| **Gene** | **Primers** | |
| --- | --- | --- |
|  |  |  |
| **ACE** | Forward | TTG CAG GCT GGC TGC TCC |
|  | Reverse | CAT TCC GCT GAT TCT GCT CTT C |
| **ANP** | Forward | CCT AAG CCC TTG TGG TGT GT |
|  | Reverse | CAG AGT GGG AGA GGC AAG AC |
| **BNP** | Forward | AGA CCC AGG CAG AGT CAG AA |
|  | Reverse | CAG CTC TTG AAG GAC CAA GG |
| **α-MyHC** | Forward | ACC GTC TGG ACG AGG AGA GCA GA |
|  | Reverse | CGT CGT GCA TCT TCT TGG CAC CAA |
| **β-MyHC** | Forward | TGC AAA GGC TCC AGG TCT GAG TCT |
|  | Reverse | GCC AAC ACC AAC CTG TGC AAG TTC |
| **Sox-6** | Forward | CTA CCC TCA GCC AAG ACA GC |
|  | Reverse | TAT GGA TTC CCA AAG CAA GC |
| **α cardiac actin** | Forward | ACT CTT GCT TGC TGA TCC AC |
|  | Reverse | GCC AAC AAT GTC CTA TCT GG |
| **α skeletal actin** | Forward | GCA TGC AGA AGG AGA TCA CA |
|  | Reverse | TTG TCG ATT GTC GTC CTC AG |
| **SRF** | Forward | ATG CCC CAT CCC TTA AAA TC |
|  | Reverse | CGC AGA AGT AGG CTT GTT CC |
| **GAPDH** | Forward | ACA CAT TGG GGG TAG GAA CA |
|  | Reverse | AAC TTT GGC ATT GTG GAA GG |
| **CREB** | Forward | CTT CCA CTT CTG CCC TCA AG |
|  | Reverse | TCC CTA AGG CAA TCA TGG AG |
