# Supplementary material for: Aldosterone Inhibits the Fetal Program and Increases Hypertrophy in the Heart of Hypertensive Mice
Source: PLoS One. 2012 May 30;7(5):e38197. doi: 10.1371/journal.pone.0038197 (PMC3364229; doi:10.1371/journal.pone.0038197)
Supplement: Table S2 — Effect of eplerenone on hemodynamic and anatomical indexes of 9-month old mice. (DOC) [file pone.0038197.s005.doc]

**Supplemental Table S2. Effect of eplerenone on hemodynamic and anatomical indexes of 9-month old mice**

WT+Eple AS+Eple Ren+Eple AS-Ren+Eple

SBP (mm Hg) 88±11 74±2 131±4 132±5

HW/BW (mg/g) 4.1±0.03 4.1±0.1 5.2±0.1 5.1±0.6 *

**Echocardiography**

LVPW (mm) 1±0.1 1.1±0.1 1.2±0.1 1.1±0.1 *

LVEDD (mm) 3.8±0.2 3.5±0.2 3.2±0.1 3.7±0.2

SF (%) 61±3 54.2±5 52.5±6.6 57±5

**mRNA expression (AU)**

ANP 0.27±0.07 0.12±0.04 0.92±0.34 1.86±0.18 *

β-MyHC 0.56±0.07 1.62±0.5 2.94±0.62 5.43±0.9 *

Sox-6 1.15±0.08 0.63±0.2 0.74±0.12 0.55±0.12 *

miR-208a 0.92±0.3 0.71±0.19 1.12±0.26 1.4±0.24 *

miR-208b 0.62±0.14 1±0.2 1.39±0.12 3.8±0.8 *

Abbreviations. SBP: Systolic blood pressure; LVPW: Left Ventricular Posterior Wall thickness; LVEDD: Left Ventricular End Diastolic Diameter; N.D.: not determined.

* p<0.05 AS-Ren vs. AS-Ren+Eple. WT: wild-type mice, AS: aldosterone-synthase overexpressing mice, Ren: renin-overexpressing mice, AS-Ren: AS and Ren crossed mice, Eple: eplerenone treatment, ns: not significant.
